# 2303 Trauma-related acute respiratory distress syndrome (ARDS) in India: Current incidence and management strategies

**DOI:** 10.1017/cts.2018.141

**Published:** 2018-11-21

**Authors:** Tessa Adžemović, Pauline Park

**Affiliations:** University of Michigan School of Medicine

## Abstract

OBJECTIVES/SPECIFIC AIMS: Aim 1: To determine the true incidence of trauma-related acute respiratory distress syndrome (ARDS) in India. We propose to perform a prospective observational study to determine the incidence of ARDS in India. Aim 2: To perform a preliminary assessment of risk factors for ARDS in the Indian trauma population. We will leverage these findings against the global ARDS data to provide a foundation for further interventional studies. Aim 3: To evaluate the current management strategies and patient outcomes from ARDS in trauma subjects admitted to the Jai Prakash Narayan Apex Trauma Center (JPNATC). These findings will identify areas in need of practice-based performance improvement in ARDS therapies in India. METHODS/STUDY POPULATION: This application proposes an observational study of trauma patients with ARDS, a population that continues to have substantial in-hospital mortality. The approximate number of ICU-admitted trauma cases for the study period is 1700. Specific data elements to be collected include patient demographics, comorbidities, mechanism of injury, Injury Severity Score, risk factors for ARDS, sequential organ failure and assessment scores, vital signs, laboratory values, and evidence-based treatments received, including mechanical ventilation and adjunctive therapies. Outcome data will include discharge location, ICU and hospital length of stay and all-cause mortality. Selection of Subjects: We will include all patients admitted to the JPNATC Trauma and Neurosurgical ICUs intubated and mechanically ventilated and meeting the definition of Berlin definition of ARDS8. We will collect data for a total of 12 months. RESULTS/ANTICIPATED RESULTS: Due to gaps in reporting, the incidence, mortality, and practice-based management algorithms applied in trauma patients suffering from ARDS in India is unknown. We hypothesize that the overall incidence of trauma-related ARDS is higher, and the fraction of patients managed with evidence-based therapies is lower than global reported averages. DISCUSSION/SIGNIFICANCE OF IMPACT: Although the true incidence of ARDS in trauma subjects in India is currently unknown, we suspect that it is much higher than reported. Such data are important in identification of resource allocation including ICU bed and mechanical ventilator availability, particularly in a resource-limited environment. This proposal will aid in the development of research infrastructure at JPNATC, contribute to capacity building, and the establishment of a Clinical Research unit at the Apex Institute. Finally, a provision to develop a consortium and trauma quality improvement program among the existing trauma centers in New Delhi to disseminate important research findings and guidance to the rest of India is a future benefit of the study.

